# Enhancing key broiler welfare indicators, meat quality, and gut microbiome composition using oxygen-enriched drinking water under commercially relevant housing conditions

**DOI:** 10.1016/j.psj.2025.105550

**Published:** 2025-07-09

**Authors:** F. Khattak, S. Galgano, R. Pearson, J.G.M. Houdijk, F. Short, A. Leigh

**Affiliations:** a Monogastric Science Research Centre, Scotland’s Rural College, Edinburgh, UK; b Oxcel, Lion Engineering Building, Gapton Hall Road, Great Yarmouth, Norfolk, UK; c UK Agri-Tech Centre, Innovation Centre, York Science Park, Heslington, York, UK

**Keywords:** oxygenated water, broiler welfare, meat quality, carcass yield, gut microbiome

## Abstract

Water is a critical nutrient in poultry production, yet its quality, particularly dissolved oxygen (DO) content, is often overlooked. This study is the first to comprehensively evaluate the impact of oxygen-enriched drinking water on broiler welfare, breast muscle myopathies, and gut microbiome composition under commercially relevant housing conditions. A total of 840 male Ross 308 broiler chicks were randomly assigned to two treatment groups (oxygenated water vs. tap water), with 12 replicate pens per treatment. Oxygenated water was enriched to a DO level of approximately 32 mg/L, compared to around 9.5 mg/L in tap water. Birds were reared to 36 days of age. The consistently high performance of both treatment groups under controlled experimental conditions is demonstrated by final body weights and feed conversion ratios surpassing Ross 308 breed standards by approximately 19–22 % from day 24 onward. Although growth performance remained unaffected under these optimal conditions, oxygenated water significantly improved welfare indicators, including feather condition, hock burn scores, and breast cleanliness (*P*< 0.05). Birds on oxygenated water also showed lower abdominal fat (−12 %) and higher thigh yield (+2.6 %) without compromising breast yield. Carcass fat deposition was significantly lower (abdominal fat pad reduced by ∼12 %), and thigh yield was higher in the oxygenated water treatment (*P* < 0.05), although overall carcass weight and breast yield were unchanged. No major differences were detected in breast meat nutrient composition (*P* > 0.05). The prevalence of white striping in breast fillets was markedly reduced in birds receiving oxygenated water, 32 mg/L indicating enhanced muscle integrity (*P* < 0.05). Metagenomic analysis revealed that some bacterial lipid metabolism pathways where differentially abundant in oxygenated-water birds. Following up on previous knowledge suggesting the interplay between lipid metabolism and broiler welfare, these findings suggest that supplementing broiler drinking water with 32 mg/L DO levels may offer a practical, non-pharmaceutical strategy to mitigate breast muscle myopathies and improve overall animal welfare and meat quality.

## Introduction

Water is often described as the most critical nutrient in poultry production, supporting a range of physiological processes essential for health and performance (Leeson and Summers, 2005). In broilers, water comprises around 70 % of body mass and is vital for thermoregulation, digestion, nutrient absorption, waste excretion, and overall metabolic function (Degen et al., 1991; Abdel-Baky et al., 2023). While considerable attention has been given to feed formulation and nutrient composition, water quality, particularly its dissolved oxygen (DO) content, remains underexplored in poultry science.

DO levels in poultry drinking water can vary widely depending on the source: well water typically contains 1–3 mg/L, surface water ranges from 5 to 14 mg/L, and tap water usually falls between 6 and 9 mg/L (Speit et al., 2002). In contrast, oxygen-enriched water, such as that generated using Micro-nano bubble technology, can achieve DO levels exceeding 30 mg/L (Li et al., 2014). These differences can influence animal physiology, particularly under intensive rearing conditions where metabolic demand is high (University of Kentucky Cooperative Extension, 1990).

Oxygen-enriched water, which is water enriched with oxygen beyond normal saturation, has gained interest as a strategy to enhance water quality and support monogastric performance. Higher DO concentrations are hypothesized to facilitate improved tissue oxygenation, reduce localized hypoxia in muscle tissue, support aerobic metabolism, and modulate oxidative stress. These physiological effects may collectively enhance growth rates, feed efficiency, and meat quality while also improving animal welfare (University of Kentucky Cooperative Extension, 1990; Gruber et al., 2005). Notably, studies in broilers have reported improved performance and physiological outcomes when provided with oxygen-enriched water containing up to 28.6 mg/L dissolved oxygen (DO) (Shin et al., 2016; Abdel-Baky et al., 2023). In laying hens, oxygenated water was administered via direct bubbling into the drinker system, although specific concentrations were not reported (ŞahanYapicier and Saatci, 2018).

Several studies in poultry and swine have reported beneficial effects of oxygenated water supplementation. In broilers, oxygen-enriched water has been associated with increased body weight, improved feed conversion ratio (up to 11.4 %), reduced abdominal fat, and enhanced immune responses (Gruber et al., 2005; Shin et al., 2016). Immunological improvements included reduced cholesterol and low-density lipoprotein levels, elevated immune globulin concentrations, and stronger innate immune responses such as increased splenic lymphocyte activity and cluster of differentiation 4 to cluster of differentiation 8 (CD4⁺:CD8⁺) ratios (Jung et al., 2012b; Shin et al., 2016). In pigs, similar effects have been observed during enteric infections, with increased cytokine expression and mononuclear cell proliferation and cytokine expression (Jung et al., 2012a). Despite these encouraging results, earlier delivery systems for oxygenated water faced practical limitations, including high energy demands, maintenance issues, particulate clogging, limited batch volumes, and oxygen loss over long distribution pipes. Nonetheless, the cumulative evidence supports its potential to enhance growth performance, carcass traits, and immunity across livestock species.

Beyond growth and immunity, oxygen-enriched water may help mitigate muscle myopathies associated with tissue hypoxia, such as white striping, which involves fibrotic and adipogenic infiltration of the breast muscle and compromises meat quality through reduced protein content and altered texture (Lee and Mienaltowski, 2023). While primarily a meat quality issue, white striping is increasingly linked to welfare concerns due to its association with oxidative stress and metabolic overload. Higher DO concentrations may reduce oxidative damage by enhancing antioxidant enzyme activity, thereby supporting muscle integrity and reducing the incidence of myopathies (Shin et al., 2016), Related conditions such as wooden breast and spaghetti meat also negatively impact both welfare and meat quality, while broader welfare indicators like footpad dermatitis, hock burn, and poor feather cover reflect environmental and physiological challenges faced by broilers under intensive production.

Oxygenated water may also benefit gut health. Increased DO levels can suppress obligate anaerobes and reduce intestinal pathogen load, creating a more favorable microbial environment. A study observed reduced coliform counts in broilers (Abdel-Baky et al., 2023), while another study reported lower *E. coli* and aerobic bacterial levels in laying hens (ŞahanYapicier and Saatci, 2018) receiving oxygenated water. However, there is limited knowledge on how these shifts affect microbiota composition at the taxonomic level or whether beneficial microbial populations are enhanced.

Despite emerging evidence on the physiological benefits of oxygen-enriched drinking water, limited research has examined its impact on welfare indicators like feather cover breast cleanliness, footpad lesions, hock burn, and meat myopathies. Moreover, no studies to date have evaluated its influence on gut microbial communities using metagenomic sequencing. This study aimed to address these knowledge gaps by assessing the effects of oxygenated drinking water on broiler welfare, carcass yield, breast muscle myopathies, and gut microbial composition. To our knowledge, this is the first integrated study to apply both phenotypic and metagenomic approaches to investigate the physiological and microbiological effects of oxygen-enriched water in broiler chickens.

## Materials and methods

### Ethical Approval

This experiment was approved by the Animal Welfare and Ethics Review Body at Scotland’s Rural College (SRUC) (AEX 2024-017 POU).

### Birds and housing

A total of 840 day-old male Ross 308 broiler chicks were obtained from a commercial hatchery and randomly assigned to two drinking water treatments in a randomized complete block design. The birds were housed at the Allermuir Avian Innovation and Skills Centre in Edinburgh, a dedicated scientific poultry research facility of SRUC designed to simulate commercial poultry production environments under controlled and bio secure conditions. The two treatments were: (1) oxygenated water and (2) regular tap water. There were 12 replicate floor pens per treatment, with 35 birds per pen (total 24 pens). Each pen measuring 4.22 m^2^ mimicking the commercial stocking density of 33 kg/m^2^. To minimise location-based bias, pens were assigned to treatments using a mirrored vertical block design. Pens 1–12 were allocated to the tap water group, and pens 13–24 to the oxygenated water group, with vertical alternation within blocks to ensure environmental conditions were balanced across the room. Each pen was deep-littered with clean wood shavings and equipped with one manual hanging feeder and a nipple drinker line containing seven nipples to ensure consistent access to feed and water. Temperature and ventilation were controlled according to Ross 308 management guidelines, and lighting was provided 23 hr light: 1 hr dark in the first week, transitioning to 18 hr light: 6 hr dark by 7 days of age onward. All birds had ad libitum access to feed and water throughout the trial period of 36 days. Light was provided by overhead LED bulbs enclosed in protective plastic covers. Lux levels were an average of 50 lux per pen, and the wavelength mix was from 420 to 780 nm. Temperature and relative humidity were measured on a daily basis and followed commercial broiler recommendations. Temperature was controlled using thermostatically regulated heating and ventilation systems to follow a standard brooding curve, starting at approximately 32°C and gradually decreasing to 20–22°C by day 36. Humidity was monitored daily and maintained between 50 and 65 % using automated ventilation misting when required. All chicks were vaccinated at the hatchery against infectious bronchitis (IB), in line with standard UK commercial practice. As the birds in this study were sourced from a commercial hatchery and had received routine vaccinations prior to placement, no further vaccinations were administered at farm level. The body weight and feed intake of each replicate pen were recorded at the start and end of each phase.

### Water Oxygenation Treatments

The oxygenated water was produced using a commercial oxygenation system (Oxcel’s oxygen-rich nano bubble generating technology) that injected 95 % (±3 %) pure oxygen into the waterlines. The system was set to oxygenate the water in a header tank twice daily (morning and afternoon) for approximately 1 hour each time. The DO levels in the drinking lines were monitored twice daily using a calibrated DO meter (Hanna H19147 DO Meter). Across the experiment, the oxygenated water lines maintained DO concentrations around 32 mg/L, whereas the regular tap water lines averaged about 9.5 mg/L DO. Both water sources were from the same supply before treatment; the only difference was the level of oxygenation. Water was supplied continuously, and the header tanks were covered to minimize gas exchange with air. Aside from oxygenation, no other water treatments (e.g., additives or sanitizers) were applied to either group. The DO levels of all water lines were recorded twice daily.

### Diets and Feeding

All birds were fed standard commercial broiler diets formulated to meet breed Ross 308 (Aviagen) nutrient recommendations. A three-phase feeding program was used: starter diet from 0 to 9 days (crumbles form), grower diet from 9 to 24 days (pellets), and finisher diet from 24 to 36 days (pellets). Feed and water were available ad libitum. The same diets were provided to both treatment groups.

### Welfare Assessment (WA)

At day 34, welfare assessment (WA) scores were recorded by a trained observer who was blinded to treatment groups to minimize inter-observer variability. All welfare measures were assessed using established scoring systems based on the RSPCA Broiler Welfare Assessment Protocol (RSPCA, 2017) and as described by (Dixon, 2020). Feather condition, particularly over the dorsal area, and breast cleanliness, defined as the presence of dirt or staining on the plumage, were scored on a scale from 0 (clean/good condition) to 3 (very dirty/poor condition), following the method outlined by (Dixon, 2020), Foot pad dermatitis and hock burn were also scored using a three-point scale, where 0 indicated no lesion or discoloration, 1 represented mild discoloration or superficial lesions, and 2 indicated deeper lesions with ulceration, hemorrhage, scabbing of significant size, or severe swelling, as previously described by (Dixon, 2020).

### Growth Performance

Birds and feed were weighed at the start and end of each feeding phase (days 0, 9, 24 and 35) to calculate body weight gain, feed intake, and feed conversion ratio (FCR) for the starter, grower, finisher, and overall 0–35 d periods. Mortality was recorded daily, and any dead birds were necropsied when possible to determine the cause of death.

#### Slaughter Measures

Due to the labor-intensive nature of the procedures, slaughter, evisceration, carcass dissection, and breast muscle evaluations for myopathies and meat composition were conducted on day 36 to ensure detailed and welfare-compliant sampling by trained staff. Four birds per pen (48 birds per treatment; 96 birds in total) were randomly selected for carcass yield evaluation and breast muscle myopathy scoring. Birds were humanely euthanized by electrical stunning, followed by exsanguination to simulate commercial processing. Carcasses were eviscerated and weighed to determine dressed (eviscerated) yield. The weights of major carcass components—including the breast muscle, thighs, drumsticks, and abdominal fat pad—were recorded and expressed as percentages of live body weight to assess yield and tissue composition. The Pectoralis major muscles (breast fillets) were scored for white striping severity on a 0–3 scale (0 = normal, 3 = severe) according to the method described by (Kuttappan et al., 2012). The wooden breast was assessed using a 2-point scale, where 0 indicated absence, and 1 indicated the presence of wooden breasts, following the approach adapted from (Sihvo et al., 2017b) and described by (Dixon, 2020). Additionally, one breast fillet sample per replicate pen was analyzed for proximate composition (moisture, crude protein, fat, and collagen) using a bench-top near-infrared (NIR) meat analyzer (FOSS).

### DNA isolation, shotgun metagenomic sequencing and bioinformatic analysis

To investigate treatment effects on gut microbiota, on day 36 at the time of slaughter, one bird per pen was humanely culled, and a 0.25 g pooled caecal sample aliquot was collected into PowerBead Pro Tube from the QIAsymphony PowerFecal Pro DNA Kit (Cat. No. 938036, QIAGEN, Hilden, Germany), and transferred to −80°C until DNA isolation. This was performed at the SRUC Biomarkers Lab (Edinburgh, UK) on QIAsymphony SP (Cat. No. 9001297, QIAGEN, Hilden, Germany), and adding 4 μl of RNase A per sample (Cat. No. 19101, QIAGEN, Hilden, Germany) after homogenization of the PowerBead Pro Tubes in a FastPrep-24™ 5 G homogenizer (MP Biomedicals, Santa Ana, CA, USA) for 55 s at 5.5 m/s.

Library preparation and sequencing was carried out following a previously described protocol (Khattak et al., 2024). In brief, genomic DNA was used to carry out quantitative and qualitative control, library preparation, and shotgun metagenomic sequencing on NovaSeq X Plus (Illumina, San Diego, California, United States) of paired-end 150 bp fragments with a target output of 12 G of raw data per sample (Biomarker Technologies (BMK) GmbH, Münster, Germany). Read quality control and Ross 308 host genome decontamination were carried out in Kneaddata, via integrating FastQC (Andrew, 2016), Trimmomatic (Bolger et al., 2014a) and Bowtie2 (Langmead and Salzberg, 2012). Taxonomy was assigned using the mpa_vJun23_CHOCOPhlAnSGB_202403 database in MetaPHlAn V3.1 pipeline (Bolger et al., 2014b; Blanco-Míguez et al., 2023), whereas functional gene tables (copies per million; CPM normalized) were created with HUMAnN 3.5 (Beghini et al., 2021), and further grouped in Kyoto Encyclopedia of Genes and Genomes (KEGG) orthologs (Kanehisa and Goto, 2000; Kanehisa et al., 2016, 2023), and pathways. Diversity analysis was carried out through calculating the richness and Shannon index (α-diversity) and the Jaccard distance and Bray-Curtis dissimilarity matrices (β-diversity) in QIIME2 (Bolyen et al., 2018) shotgun distribution (v2024-5).

### Statistical analysis

All growth performance and welfare assessment (WA) score data were analyzed using general analysis of variance (ANOVA) in Genstat (23rd Edition). The statistical model included replicate pen and block as random effects, with water treatment (tap vs. oxygenated) as the fixed effect. The experimental unit was the pen for growth performance data and the individual bird for WA scores. A priori power analysis indicated that 10–12 replicates per treatment would provide ≥80 % power to detect a 3-point difference in FCR (1.653 vs. 1.603) over 42 days (SD = 0.08, α = 0.05, two-sided t-test). Homogeneity of variance across treatment groups was assessed visually using histograms and boxplots and formally using Bartlett’s test. All data met assumptions of normality and, therefore were not transformed. No outliers were removed from the dataset. Statistical significance was assessed at the 5 % level (*P* < 0.05) using approximate F-tests.

For metagenomics, the R v4.3.3 (R Core Team, 2022) package *lme4* (Bates et al., 2015) was used to carry out the linear mixed model (LMM) with treatment (or test variable) as fixed effect and block as a random effect, thanks to which α-diversity and taxonomical data were analyzed and the *P* value was calculated with a type III ANOVA with Satterthwaite's degrees of freedom method, using the R package *lmerTest* (Kuznetsova et al., 2017). Additionally, the false discovery rate (type I error) under repeated testing was also calculated for the taxonomical data, which allowed to calculate the Q value through the package fuzzySim (Barbosa, 2015). The R package MaAsLin2 (Mallick et al., 2021) was used to carry out the multivariable associations through performing a Compound Poisson Linear Model (CPLM). This allowed to carry out multiple comparison testing on the gene families and pathways feature tables. Thus, for both the analyses, only the comparison with both *P* and *Q* values < 0.05 were considered significant.

## Results

### Water Oxygenation Levels

The trial was conducted from mid-July to late August in a temperature-controlled research broiler facility, which maintained consistent ambient conditions and minimized external seasonal effects. The DO levels in the two water treatments remained consistently distinct throughout the trial. The oxygenated water maintained an average DO concentration of approximately 32 ± 1.55 mg/L (min: 28 mg/L, max: 36 mg/L), while the tap water averaged 9.5 ± 0.92 mg/L (min: 6.3 mg/L, max: 10.5 mg/L). These values confirm that the oxygenation system effectively sustained a DO concentration that was more than three times higher in the treated water than in the tap water, demonstrating a consistent separation between the two treatment groups.

### Growth Performance

Growth performance data revealed no significant differences (*P* > 0.05) between the oxygenated water and tap water groups during the starter, grower, finisher, or overall (0–35 d) phases (Table 1). Final body weights at day 35 were also not significantly different between treatments (*P* > 0.05). Both groups exhibited high growth rates, with average body weights exceeding the Ross 308 performance objectives (breed targets) by approximately 19–22 % from day 24 onward. Overall mortality through day 35 was low (2.1 %) and did not differ between treatments. The predominant cause of mortality was sudden death syndrome “flip-over”, with six birds affected in each treatment group. A small number of isolated cases of leg deformities, poor growth, or physical anomalies (e.g., a single case of a twisted beak) were observed, with no apparent association with water treatment.

**Table 1:** Effects of oxygenated drinking water compared to tap water on broiler performance during starter, grower and finisher phase.

Treatment	Starter Phase (d 0-9)			Grower Phase (d 9-24)			Finisher Phase (d 24-35)			Overall performance (d 0-35)		
	ADG	AFI	F:G	ADG	AFI	F:G	ADG	AFI	F:G	ADG	AFI	F:G
	(kg/bird/d)		(kg:kg)	(kg/bird/d)		(kg:kg)	(kg/bird/d)		(kg:kg)	(kg/bird/d)		(kg:kg)
Tap water	0.027	0.027	1.015	0.085	0.107	1.255	0.129	0.186	1.444	0.080	0.105	1.309
Oxygenated water	0.027	0.028	1.010	0.084	0.106	1.257	0.125	0.184	1.467	0.079	0.104	1.318
SED	0.000	0.000	0.008	0.001	0.001	0.007	0.002	0.001	0.016	0.001	0.001	0.004
P-Value	0.206	0.082	0.585	0.252	0.305	0.713	0.112	0.135	0.167	0.125	0.277	0.067

*n* = 12 pens/treatment.

SED = Standard errors of difference of means.

ADG = Average daily gain.

AFI = Average daily feed intake.

F:*G* = Feed to gain ratio.

P-Value = Significance level (*P* < 0.05).

### Carcass Yield and Meat Composition and myopathies

At processing, there were no significant differences (*P* > 0.05) in live bird weight, eviscerated carcass weight, or the yield of major cuts (breast and drumstick weights as a percentage of live weight) between the two treatment groups, although birds given oxygenated water tented to have slightly higher values across these measures (Table 2). However, birds receiving oxygenated water exhibited significantly lower abdominal fat yield (11.6 % reduction) and significantly higher thigh yield (2.6 % increase) compared to the tap water group (*P* < 0.05; Table 2). These results suggest a modest shift in nutrient partitioning in the oxygenated water group, characterized by reduced fat deposition and a slight increase in lean tissue development in the thighs.

**Table 2:** Effects of oxygenated drinking water compared to tap water on carcass traits of broilers processed at day 36.

Treatment	Live Body weight	Carcass weight	Carcass	Breast	Drum	Thigh	Abdominal Fat
	(kg)	(kg)	(%)	(%)	(%)	(%)	(%)
							
Tap water	3.106	2.229	71.750	30.270	13.117	15.610	1.247
Oxygenated water	3.183	2.888	71.880	30.570	13.396	16.030	1.117
SED	0.049	0.0371	0.316	0.339	0.1898	0.230	0.060
P-Value	0.120	0.112	0.678	0.385	0.145	0.070	0.033

*n* = 72 samples/treatment group.

Live weight used here is of the birds used in processed data:.

Carcass (%) = Carcass weight/live body weight * 100.

Breast (%) = Breast weight/carcass weight * 100.

Drum (%) = Drum weight/carcass weight * 100.

Thigh (%) = Thigh weight/carcass weight * 100.

Fat (%) = Fat weight/carcass weight * 100.

P-Value = Significance level (*P* < 0.05).

The nutrient composition of the breast meat revealed no significant treatment effects (*P* > 0.05). Moisture, crude protein, fat, and collagen content of breast fillets were statistically similar between birds receiving oxygenated and tap water (Table 3). There was a non-significant trend toward higher intramuscular fat in the oxygenated water group (mean 2.08 % vs. 1.69 % in tap water; *P* = 0.072).

**Table 3:** Effects of oxygenated drinking water compared to tap water on breast nutrient composition using Foss Analysis.

Treatment	Moisture %	Protein %	Collagen %	Fat %
Tap water	75.46	20.33	0.994	1.695
Oxygenated water	75.06	20.15	1.034	2.084
SED	0.517	0.227	0.693	0.196
P-Value	0.453	0.440	0.098	0.072

*n* = 12 breasts/treatment.

SED = Standard errors of difference of means.

P-Value = Significance level (*P* < 0.05).

The distribution of breast muscle myopathy scores is summarized in Table 4. Birds receiving oxygenated water had a significantly higher proportion of fillets scored as normal (white striping score 0) than the tap water group (40 % vs. 27 %; *P* = 0.016). Although not statistically significant, there was a numerical reduction in the proportion of fillets with mild (score 1) and moderate (score 2) white striping in the oxygenated group compared to the tap water group (50 % vs. 58 % for score 1; 9 % vs. 15 % for score 2, respectively; *P* > 0.05).

**Table 4:** Effect of oxygenated drinking water compared to tap water on mean percentage of each breast striation and wooden breast score.

Treatment	Breast Striation Score			Wooden Breast Score	
	0	1	2	0	1
Tap water	0.27	0.58	0.15	0.833	0.125
Oxygenated water	0.40	0.50	0.09	0.865	0.135
SED	0.053	0.063	0.049	0.052	0.045
P-Value	0.016	0.194	0.300	0.549	0.820

*n* = 12 breasts/treatment.

Breast Striations (White Striping) = Each breast was assessed on a 3 point scale (0-2) where:.

0 = no striping,.

1 = moderate degree of striping,.

2 = severe degree of striping.

Wooden Breast—Each breast was assessed on a 2 point scale (0-1) where:.

0 = absence of wooden breast,.

1 = presence of wooden breast.

P-Value = Significance level (*P* < 0.05).

Wooden breast scores were not significantly affected by water treatment (*P* > 0.05, Table 4). The majority of fillets in both groups showed no signs of wooden breast (score 0), with 86.5 % in the oxygenated water group and 83.3 % in the tap water group (*P* = 0.549). A small proportion of fillets showed the presence of wooden breast (score 1), with no difference between treatments (13.5 % *vs*. 12.5 %, *P* = 0.820).

### Welfare Assessments (WA)

**Fig 1:**
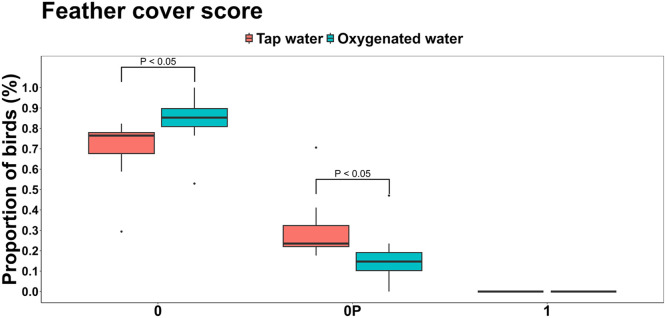
Effects of oxygenated drinking water compared to tap water on the mean proportions of feather cover scores.

**Fig 2:**
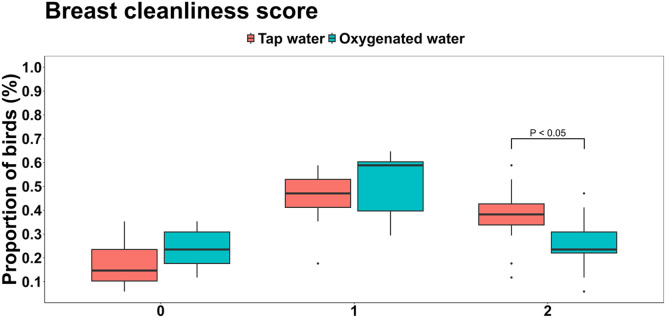
Effects of oxygenated drinking water compared to tap water on breast cleanliness scores.

Several welfare-related differences were observed at day 36. As shown in Figure 1, a significantly higher proportion of birds in the oxygenated water group had full feather cover (score 0) compared to the tap water group (84.3 % vs. 70.1 %; *P* = 0.024). Conversely, the incidence of slightly patchy feathering on the sides (score OP) was 47.6 % lower in the oxygenated group (15.7 % vs. 29.9 %; *P* = 0.024). Water treatment had no significant effect on breast cleanliness scores 0 (clean plumage) or 1 (slightly dirty plumage) (*P* > 0.05); however, the proportion of birds with a score of 2 (large dirty patches) was significantly lower in the oxygenated group, representing a 31.6 % reduction compared to the tap water group (Figure 2).

**Fig 3:**
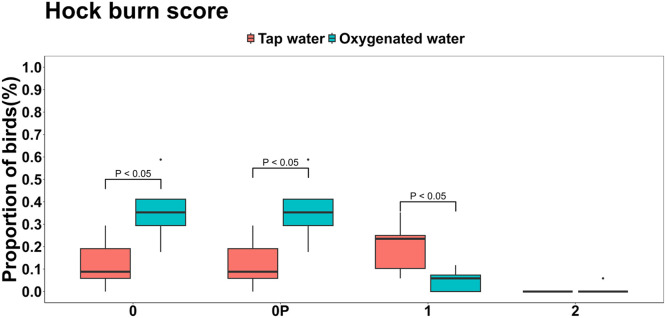
Effects of oxygenated drinking water compared to tap water on mean proportions of hock burn scores.

**Fig 4:**
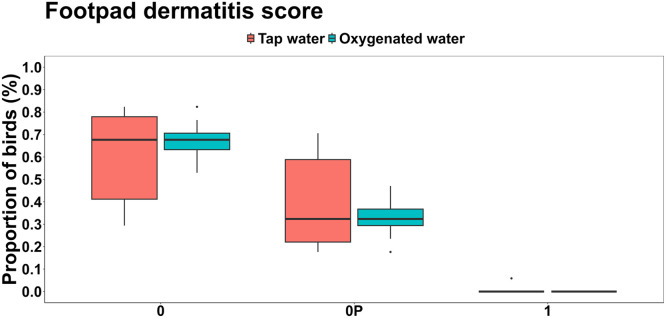
Effects of oxygenated drinking water compared to tap water on mean proportions of footpad dermatitis score.

Hock burn scores were significantly improved in birds receiving oxygenated water (Figure 3). A higher proportion of birds in this group had a score 0 (no lesions) compared to the tap water group (0.373 vs. 0.123; *P* < 0.001), while a score 1 (mild lesions) was significantly less frequent in the oxygenated group (0.054 vs. 0.201; *P* = 0.004). Only one bird in each group exhibited early signs of score 2 (larger surface area affected without lesions or hemorrhage), with no significant difference between treatments (*P* = 0.166). However, footpad dermatitis scores did not differ significantly between treatments (*P* > 0.05; Figure 4). A slightly higher percentage of birds given oxygenated water had healthy footpads (score 0) compared to those receiving tap water (66.7 % vs. 60.8 %). Few birds showed mild lesions (score 1), and no birds in either group exhibited severe lesions (score 2).

### Metagenomic analysis

**Fig 5:**
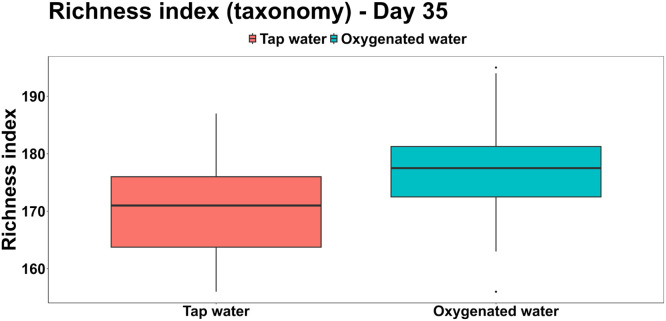
Richness index (alpha-diversity) calculated for the taxonomical features across the two treatments.

**Fig 6:**
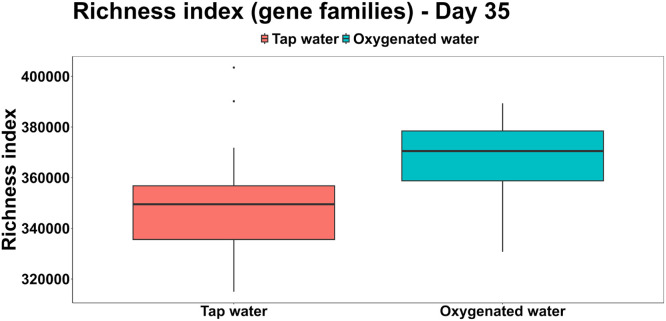
Richness index (alpha-diversity) calculated for the functional genes (Uniref90 gene families) across the two treatments.

All the samples were within the target yield of 12 Gb, with an average of 45,000 reads per sample. The α-diversity of the taxonomic features, as assessed using Shannon and richness inices, did not differ significantly between treatments. However, the richness appeared modestly higher in the oxygenated water group (177.04 ± 9.19) compared to the tap water group (170.04 ± 8.84) (Figure 5). In contrast, when richness was calculated based on gene family counts, a significant increase was observed in the oxygenated water group (367,749.58 ± 16,204.48 features) relative to the tap water group (348,909.54 ± 20,931.00 features; F(1,46) = 12.16, *P* < 0.01) (Figure 6).

**Fig 7:**
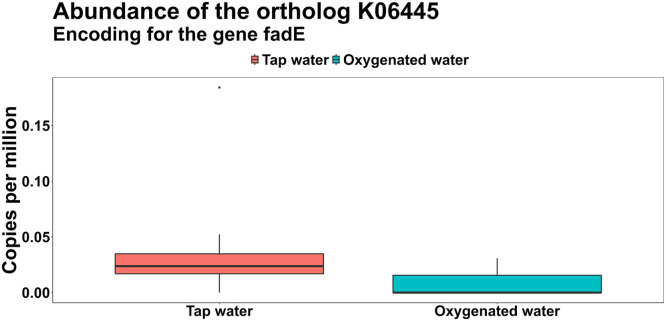
Normalized abundance in copies per million of the ortholog *fadE* (K06445), encoding for the cyl-coenzyme A dehydrogenase gene, across the treatments.

**Fig 8:**
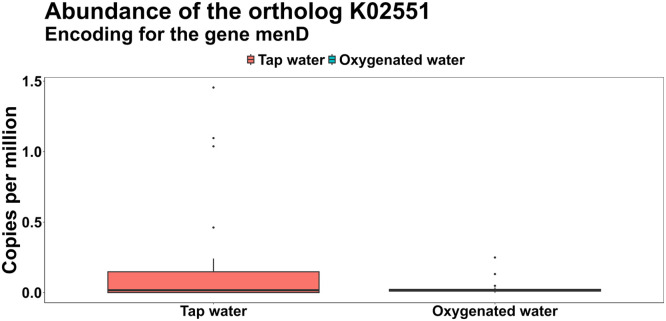
Normalized abundance in copies per million of the ortholog *menD* (K02551), encoding for the 2-succinyl-5-enolpyruvyl-6‑hydroxy-3-cyclohexene-1-carboxylate synthase gene across the treatments.

Cumulatively, Firmicutes was the most abundant phylum in the caeca of all the birds (81.45 % ± 6.56 %), followed by Bacteroidota (8.34 % ± 3.44 %), Actinobacteria (8.82 % ± 5.66 %), Proteobacteria 90.92 % ± 0.47 %), Tenericutes (0.39 % ± 0.51 %) and Verrucomicrobia (0.07 % ± 0.21 %), however no significant differences were found in the relative abundance of these phyla across the two treatments (Supplementary Information 1). In parallel, the ten most abundant genera, cumulatively, were *Faecalibacterium* (9.36 % ± 3.29 %), *Alistipes* (8.06 % ± 3.36 %), *Bifidobacterium* (7.77 % ± 5.28 %), *Lactobacillus* (6.11 % ± 4.97 %), *Lachnoclostridium* (5.79 % ± 2.04 %), genus level genome bin (GGB) 3609 (5.21 % ± 3.6 %), GGB9168 (4.12 % ± 4.56 %), GGB45537 (3.85 % ± 1.17 %), *Blautia* (3.09 % ± 2.38 %) and unclassified *Eubacteriaceae* (2.78 % ± 1.51 %). The complete Genus level composition across the two treatments is provided in Supplementary Information 2. The annotated gene families were further grouped into 8,954 KEGG unstratified orthologs (KOs), and notably, two orthologs—*fadE* (K06445) and *menD* (K02551)—were significantly less abundant in the oxygenated water group (Figure 7 and Figure 8). The *fadE* gene encodes acyl-CoA dehydrogenase, an enzyme involved in the initial steps of fatty acid β-oxidation, while *menD* is involved in the biosynthesis of menaquinone (vitamin K2), which plays a key role in microbial respiration.

## Discussion

This study evaluated the effects of oxygen-enriched drinking water (32 mg/L DO) on broiler chickens, with a focus on growth performance, welfare, carcass traits, and muscle myopathies. Significant impacts on the latter three were observed. However, despite numerical trends favoring the oxygenated group, oxygenation did not significantly improve growth performance, contrary to previous reports(Shin et al., 2016; Abdel-Baky et al., 2023). One likely reason is that birds in both groups performed exceptionally well, surpassing Ross 308 breed targets by 19–22 % from day 24 onwards. These findings align with studies showing negligible growth benefits of increased DO under non-stressful conditions (Zimmermann et al., 1991; Dinçer et al., 2007). This suggests that supplemental DO in drinking water does not consistently enhance growth when oxygen availability is not a limiting factor. However, under commercial conditions involving stressors such as heat, poor ventilation, or disease, oxygenated water may offer more pronounced benefits (Shin et al., 2016). Although differences in growth performance were not statistically significant, numerical trends suggest potential biological relevance. Broilers given oxygenated water showed a slight improvement in overall feed-to-gain ratio (1.318 vs. 1.309; *P* = 0.067). Average daily gain (ADG) and average feed intake (AFI) were similar between groups across all phases. From a production standpoint, even small improvements in feed:gain can yield economic benefits, given that feed accounts for 60–70 % of total costs. The observed trend toward improved efficiency without compromising growth supports further evaluation in larger-scale trials to confirm practical value.

Variability in the outcomes of studies investigating oxygen-enriched water may, in part, reflect differences in oxygen delivery methods (Zimmermann et al., 1991; Gruber et al., 2005; Jung et al., 2012a; Shin et al., 2016; Abdel-Baky et al., 2023). These studies reflect differences in technology, DO levels, and application contexts (e.g. pigs vs. poultry, stressed vs. non-stressed environments), which may contribute to inconsistent results across the literature. In the present study, a nanobubble system (Oxcel technology) was used to administer DO. This system generates neutrally buoyant nanobubbles that remain suspended in water for at least 24 hours post-oxygenation under typical poultry housing conditions, even in the presence of organic matter such as biofilm residues, feed dust, or environmental particulates, thereby maintaining elevated DO levels more consistently than traditional microbubble systems. The technology incorporates a centralized injection unit connected to the farm’s main water supply and includes automated controls for real-time monitoring and adjustment of DO concentrations. In this study, DO levels remained stable and close to 32 mg/L throughout the experimental period, confirming the reliability and uniform distribution of oxygen-enriched water. These features collectively support the feasibility of consistent DO delivery at a field scale within commercial poultry production systems.

Crucially, our study demonstrated significant improvements in several broiler welfare indicators among birds receiving oxygen-enriched water. These birds showed better feather coverage, cleaner plumage, and fewer hock burns, which are physical traits commonly associated with comfort, mobility, and reduced environmental or metabolic stress (RSPCA, 2017; Dixon, 2020). Improved feather condition may reflect better thermoregulation and nutrient status, while lower hock burn incidence indicates reduced contact dermatitis and potentially enhanced immune competence (Castilla et al., 2012; Růžička et al., 2021). We hypothesize that increased oxygen availability enhanced peripheral tissue oxygenation and healing capacity, contributing to improved integument condition. Although we did not measure direct physiological or behavioral stress markers, these visual indicators are widely accepted proxies of broiler welfare. Notably, the variance in footpad scores was substantially lower in the oxygenated water group (0.0078) compared to the tap water group (0.0411), suggesting more uniform welfare outcomes. This reduced variability may reflect a stabilizing effect of oxygenated water on litter quality or physiological resilience, potentially resulting in fewer individual birds experiencing poor welfare conditions. The prevalence of key animal welfare indicators in commercial broiler systems varies but typically ranges between 15 and 35 % for footpad dermatitis (FPD), 5–20 % for hock burns, and 2–15 % for lameness, depending on genetics, litter conditions, and management (Haslam et al., 2007; Bassler et al., 2013). In our study, FPD and hock burn scores remained within or below these ranges across treatments, indicating no upward deviation from expected prevalence levels.

Improvements in carcass traits observed in birds receiving oxygen-enriched water suggest beneficial shifts in metabolic partitioning. Specifically, the significant reduction in abdominal fat and concurrent increase in thigh muscle yield align with earlier findings indicating that higher DO intake may enhance lean tissue accretion and reduce fat deposition (Shin et al., 2016; Abdel-Baky et al., 2023). These effects are likely mediated by improved aerobic metabolism, which promotes energy utilization through oxidative pathways rather than lipogenesis (Kikusato and Toyomizu, 2013). Thigh muscles, being more oxidative and richly vascularized than breast muscles, are especially responsive to enhanced oxygen availability, which may stimulate protein synthesis while limiting catabolic processes (Lee and Mienaltowski, 2023). From a production standpoint, this repartitioning toward leaner carcasses and higher-value cuts offers both economic and consumer health benefits.

Our metagenomic analysis provides further support for these physiological changes. A reduced abundance of microbial genes such as fadE (acyl-CoA dehydrogenase) and menD (involved in menaquinone biosynthesis) suggests suppression of fatty acid β-oxidation and microbial vitamin K₂ synthesis, potentially reflecting altered gut redox balance under elevated oxygen conditions (Rivera-Chávez and Bäumler, 2015; Ren et al., 2020). These functional shifts may contribute to more efficient nutrient utilization and reduced microbial competition for host energy substrates.

One of the most novel findings in this study was the reduced severity of white striping myopathy in the oxygenated water group. White striping is a common degenerative condition in rapidly growing broilers, characterized by intramuscular fat and fibrosis, and is strongly associated with localized muscle hypoxia and oxidative stress (Lee and Mienaltowski, 2023). This condition is highly relevant in commercial practice, with reported prevalence rates ranging from 50 % to over 90 % in high-yield broiler strains depending on age, genotype, and management practices (Kuttappan et al., 2012; Lee and Mienaltowski, 2023). In our study, approximately 60 % of fillets from the control group (tap water) exhibited white striping scores of 1 or 2, compared with 59 % in the oxygenated group. Importantly, the proportion of fillets with no observable lesions (score 0) increased from 27 % in control to 40 % in the oxygenated group, indicating a meaningful reduction in severity. It can be hypothezied that increased DO availability enhanced tissue oxygenation in the Pectoralis major, thereby reducing hypoxic damage and limiting fat infiltration. Previous work has shown that oxygen-rich water elevates antioxidant enzyme activity and reduces lipid peroxidation in poultry, supporting the hypothesis that better oxygenation limits oxidative muscle damage (Shin et al., 2016). Interestingly, while white striping severity was reduced, no significant differences were observed in the incidence of wooden breast. This discrepancy may reflect differences in the pathogenesis and severity of the two myopathies. White striping is generally considered a milder condition, more sensitive to improvements in metabolic and vascular function, whereas wooden breast involves more extensive fibrosis and muscle rigidity, potentially requiring longer or more intense intervention to observe effects (Petracci et al., 2015; Sihvo et al., 2017a; Lee and Mienaltowski, 2023). Thus, the lack of response in wooden breast incidence may not negate the beneficial role of oxygen-enriched water but rather highlight the differing biological thresholds of these conditions. To our knowledge, this is the first report demonstrating that drinking water oxygenation can reduce the incidence of breast muscle myopathies in broilers, offering practical and economic benefits by improving meat quality and reducing carcass downgrading (Kuttappan et al., 2012; ŞahanYapicier and Saatci, 2018).

Our achieved DO level in the oxygenated water averaged 32.5 mg/L across the study period, with daily measurements ranging from 27.9 to 36.2 mg/L. These concentrations substantially exceed those used in many earlier trials. Earlier studies administering lower DO (10–15 mg/L) under standard conditions often reported little to no response, whereas trials using ≥ 25 mg/L or higher, particularly under environmental or physiological stress, demonstrated more consistent benefits (Zimmermann et al., 1991; Shin et al., 2016; Abdel-Baky et al., 2023). Our findings reinforce that DO concentrations above 25 mg/L are safe and potentially advantageous. Given the stable delivery of elevated DO under commercial summer conditions, future studies should explore the dose-response relationship under varying environmental or nutritional stressors to strategically tailor DO technology deployment for optimized cost-effectiveness.

The cecal microbiome analysis revealed functional changes in response to oxygenated water. The enrichment of genes involved in fatty acid biosynthesis, pyrimidine metabolism, phosphonate catabolism, and urea hydrolysis suggest a more metabolically active and host-supportive microbial community (Pan and Yu, 2013; Oakley et al., 2014). These functions contribute to short chain fatty acid production, nitrogen recycling, and phosphorus utilization, all beneficial to gut and systemic health (Koh et al., 2016; Rubio, 2019). Enhanced urea hydrolysis, for instance, may have improved amino acid availability, contributing to better feathering. Reduced abundance of genes involved in fatty acid degradation and menaquinone biosynthesis further indicates a shift away from strictly anaerobic or energy-competing microbes (LeBlanc et al., 2013; Ren et al., 2020). While the decrease in microbial vitamin K₂ production is unlikely to affect bird health due to dietary supplementation, the overall shift supports improved nutrient utilization and gut functionality under oxygen-enriched conditions.

From a practical standpoint, oxygenated water is a promising, residue-free strategy to enhance welfare and carcass quality in broiler production without compromising growth. This is particularly relevant for antibiotic-free or high-welfare production systems. Although installation and operation of oxygenation systems require an upfront investment, the resulting improvements, such as reduced myopathies, leaner carcasses, and improved welfare indicators, could offset costs through enhanced product quality and market value. However, it should be noted that the current study was conducted on male broilers from a single genetic line, under controlled housing conditions. To confirm and extend these findings, further research is needed in female birds, alternative broiler genotypes, and diverse housing systems, including organic and slower-growing production models. Future research should aim to define the minimum effective DO concentration, assess responses under commercial stress conditions, and evaluate long-term economic returns across multiple production cycles and poultry types.

## Conclusion

This study demonstrates that supplementing broiler drinking water with elevated dissolved oxygen (32 mg/L) can enhance carcass composition, reduce the severity of breast muscle myopathies, and improve several welfare indicators, without compromising growth performance. Although differences in performance metrics such as feed conversion ratio were not statistically significant, observed trends suggest potential biological relevance and economic value. The consistent and stable delivery of oxygen using nanobubble technology underscores the practical feasibility of this approach under commercial poultry housing conditions. Notably, oxygenated water reduced the severity of white striping in breast muscles, improved feather and hock condition, lowered abdominal fat, and increased thigh muscle yield, the traits of growing interest to both consumers and producers. Functional gut microbiome analysis supports these findings, revealing beneficial shifts in microbial metabolic activity that likely contribute to improved nutrient partitioning and host health. While growth performance remained unchanged, the improvements in carcass quality and welfare support the use of oxygenated water as a sustainable, non-antibiotic intervention. Future research should aim to validate these findings under typical commercial stressors such as pathogen exposure, where oxygen enriched water may yield more pronounced benefits. Dose response trials are needed to determine the minimum effective DO concentration for cost effective application. Additionally, studies involving female birds, alternative genotypes, slower growing strains, and varied housing systems including organic or antibiotic free operations will help assess broader applicability. Longitudinal trials across multiple production cycles are also recommended to evaluate sustained performance benefits and economic returns. Finally, investigations integrating physiological, immunological, and behavioral endpoints would enhance understanding of how oxygen supplementation influences broiler health, welfare, metabolism, and resilience.

## Funding

The work was funded by Innovate UK (Project number10107943) under the competition “Launchpad: Agri-tech and food tech in Eastern England”. SRUC receives support from Scottish Government (RESAS).

## Disclosures

AL and RP are the manufacturers of the Oxcel technology system used in this study. As such, Oxcel Ltd provided the water enhancement system used in this study. As co-authors, AL and RP had some influence over the study design, however, they had a smaller role to play in the decision to publish or in the preparation of the manuscript.

The remaining authors declare that they have no conflict of interest.

## Appendix Supplementary materials

Image, application 1

Image, application 2

## Footnotes

Supplementary material associated with this article can be found, in the online version, at doi:10.1016/j.psj.2025.105550.
